# Comprehensive Assessment of Biventricular and Biatrial Mechanics in Patients with Extracardiac Sarcoidosis Without Fibrotic Pulmonary Involvement

**DOI:** 10.3390/jcm15051743

**Published:** 2026-02-25

**Authors:** Andrea Sonaglioni, Antonella Caminati, Federico De Cesco, Alessandro Lucidi, Gian Luigi Nicolosi, Massimo Baravelli, Michele Lombardo, Sergio Harari

**Affiliations:** 1 Division of Cardiology, IRCCS MultiMedica, 20123 Milan, Italy; massimo.baravelli@multimedica.it (M.B.); michele.lombardo@multimedica.it (M.L.); 2 Semi-Intensive Care Unit, Division of Pneumology, IRCCS MultiMedica, 20123 Milan, Italy; antonella.caminati@multimedica.it (A.C.); federico.decesco@multimedica.it (F.D.C.); alessandro.lucidi@multimedica.it (A.L.); sergio.harari@unimi.it (S.H.); 3 Division of Cardiology, Policlinico San Giorgio, 33170 Pordenone, Italy; gianluigi.nicolosi@gmail.com; 4 Department of Clinical Sciences and Community Health, Università di Milano, 20122 Milan, Italy

**Keywords:** sarcoidosis, subclinical myocardial dysfunction, global longitudinal strain, left atrial reservoir strain, right atrial reservoir strain, apical sparing pattern

## Abstract

**Background:** Speckle-tracking echocardiography (STE) has been increasingly used to uncover subtle cardiac dysfunction in patients with extracardiac sarcoidosis (ECS) who show no clinical evidence of heart disease. However, prior investigations were mostly retrospective, methodologically heterogeneous, and focused primarily on left ventricular (LV) function. We conducted a prospective study to provide a broader evaluation of myocardial deformation across both ventricles and atria in ECS without fibrotic pulmonary involvement. **Methods:** Forty-one patients with ECS (mean age 57.4 ± 10.2 years; 58.5% male) and 30 age- and sex-matched controls without ECS and without known structural heart disease (58.5 ± 11.1 years; 53.3% male) were enrolled. All participants underwent conventional transthoracic echocardiography (TTE) supplemented by comprehensive STE analysis of ventricular and atrial function. Subclinical myocardial dysfunction was defined as LV global longitudinal strain (GLS) less negative than −20%, and potential predictors were analyzed. **Results:** Standard TTE did not show echocardiographic features suggestive of overt infiltrative cardiomyopathy but revealed higher E/average e′ ratios in the ECS group, suggesting subtle diastolic dysfunction. While traditional indices of biventricular systolic function remained preserved, STE demonstrated significant reductions in LV-GLS, LV global circumferential strain, right ventricular-GLS, and both left and right atrial reservoir strain. Multivariate analysis identified disease duration as the sole independent determinant of LV-GLS impairment (OR 2.26, 95%CI 1.10–4.65; *p* = 0.03). A disease duration of ≥4.5 years predicted abnormal GLS with 88% sensitivity and 75% specificity (AUC 0.89; 95%CI 0.76–1.00). **Conclusions:** ECS without fibrotic pulmonary involvement is associated with early impairment of biventricular and biatrial strain despite preserved conventional function. The extent of dysfunction correlates strongly with disease duration, underscoring the value of STE for early detection and monitoring.

## 1. Introduction

Sarcoidosis is a systemic inflammatory disease of uncertain etiology characterized by the formation of non-caseating granulomas within involved tissues and associated with significant morbidity [1]. Although pulmonary and mediastinal involvement represents the most frequent manifestation—affecting approximately 80% of patients—granulomatous lesions may also occur in other organs, including the skin, parotid glands, spleen, liver, central nervous system, bone, eyes, lymphatic system, and myocardium [2].

The reported prevalence of cardiac involvement in systemic sarcoidosis ranges widely, from approximately 3.7% to 54.9%, largely depending on the diagnostic criteria applied and the characteristics of the studied populations [3,4]. Notably, up to one-third of patients with myocardial involvement may remain asymptomatic, whereas clinically manifest cardiac sarcoidosis (CS) is observed in only about 5% of cases [5]. When symptomatic, CS most commonly presents with atrioventricular conduction disturbances, ventricular arrhythmias, or heart failure [6]. Given that cardiac involvement represents one of the principal causes of sarcoidosis-related mortality [7], timely identification of myocardial granulomatous infiltration is of paramount clinical importance.

Screening for CS generally begins with a clinical evaluation, including focused history-taking, physical examination, and a standard 12-lead electrocardiogram (ECG) [8]. However, ECG abnormalities lack specificity for CS and therefore cannot reliably confirm or exclude cardiac involvement when used in isolation [9].

Advanced imaging techniques, such as late gadolinium enhancement cardiac magnetic resonance (LGE-CMR) and 18F-fluorodeoxyglucose positron emission tomography (FDG-PET), offer superior diagnostic performance in terms of sensitivity and specificity for detecting CS [10,11]. Nevertheless, their routine use as universal screening tools is limited by several factors, including cost and accessibility (particularly for CMR), contraindications related to patient characteristics (e.g., severe obesity, impaired renal function, or the presence of intracardiac devices in the case of CMR), and radiation exposure in the case of PET imaging. Endomyocardial biopsy is also unsuitable for screening purposes, as it is invasive and characterized by limited sensitivity due to the patchy distribution of myocardial lesions [8].

Conventional two-dimensional (2D) transthoracic echocardiography (TTE), in combination with ECG and clinical assessment, is therefore recommended as an initial screening modality in patients with biopsy-proven extracardiac sarcoidosis (ECS) [12]. TTE is broadly available, noninvasive, and more cost-effective than CMR or FDG-PET. However, traditional echocardiographic parameters of systolic and diastolic function demonstrate limited sensitivity and specificity for detecting early myocardial involvement [13].

Technological advances in cardiac imaging have led to the development of two-dimensional speckle-tracking echocardiography (2D-STE), an angle-independent technique capable of quantifying both global and regional myocardial mechanics by measuring deformation (strain) and strain rate [14]. This Method Enables The Detection Of Subtle Myocardial Dysfunction, Typically Manifested By A Reduction In Left Ventricular (LV) Global Longitudinal Strain (GLS) To Less Negative Than −20%, Even In The Presence Of A Preserved Left Ventricular Ejection Fraction (LVEF ≥ 55%) [15].

During the past decade, multiple studies [16,17,18,19,20,21,22,23,24,25,26,27,28] have investigated ECS patients without overt cardiac disease using conventional 2D-TTE complemented by 2D-STE to identify early myocardial impairment. However, these studies were heterogeneous in design, frequently retrospective, and predominantly centered on LV-GLS, without providing a comprehensive evaluation of deformation parameters across all four cardiac chambers.

On this basis, we conducted a prospective case–control study to compare biventricular and biatrial myocardial deformation indices in consecutive patients with ECS without fibrotic pulmonary disease with those of age- and sex-matched controls without ECS or structural heart disease. In addition, we explore potential pathophysiological mechanisms that may underlie subclinical myocardial dysfunction in this population.

## 2. Materials and Methods

### 2.1. Study Population

This prospective study included a consecutive series of patients with extracardiac sarcoidosis (ECS) referred to the Division of Pneumology at San Giuseppe MultiMedica IRCCS Hospital between 13 January 2025 and 19 May 2025. The ECS cohort was compared with age- and sex-matched individuals without ECS and without evidence of structural heart disease [29]. Control subjects were recruited from the Outpatient Cardiology Division of the same institution.

The diagnosis of ECS was established based on chest computed tomography (CT) findings and bronchoscopic evaluation, with final confirmation obtained through multidisciplinary team discussion. Histopathological evidence of non-caseating granulomas in extracardiac tissue samples collected via bronchoscopy was required for study inclusion, in accordance with current international diagnostic recommendations [30,31].

Eligible patients fulfilled the following criteria: confirmed ECS according to established guidelines [30,31], pulmonary sarcoidosis stages 0–III, and clinical stability for at least three months prior to enrollment.

Exclusion criteria comprised: clinically manifest cardiac sarcoidosis; advanced fibrotic pulmonary sarcoidosis (stage IV); hemodynamic instability at the time of evaluation; inadequate acoustic windows preventing accurate delineation of ventricular and atrial endocardial borders; and unwillingness to participate.

Baseline clinical data were retrieved from medical records and included demographic and anthropometric variables (age, sex, body surface area, body mass index); cardiovascular risk profile (smoking status, arterial hypertension, type 2 diabetes mellitus, dyslipidemia); prior cardiovascular or cerebrovascular events (including coronary revascularization, stroke, or transient ischemic attack); electrocardiographic parameters (cardiac rhythm, heart rate, intraventricular conduction abnormalities); major comorbid conditions [malignancy, chronic obstructive pulmonary disease (COPD), obstructive sleep apnea syndrome (OSAS), gastroesophageal reflux disease (GERD), rheumatoid arthritis, hypothyroidism, mixed anxiety–depressive disorder]; laboratory measurements [complete blood count including hemoglobin, red cell distribution width (RDW), neutrophil-to-lymphocyte ratio (NLR); serum creatinine with estimated glomerular filtration rate (eGFR) calculated as previously described [32]; fasting glucose and total cholesterol]; and ongoing pharmacological treatments.

During the baseline visit, all ECS participants underwent comprehensive respiratory and cardiovascular assessment, including spirometry, measurement of diffusing capacity for carbon monoxide (DLCO), six-minute walk test (6MWT), blood pressure measurement, standard 12-lead electrocardiography, and detailed transthoracic echocardiography with two-dimensional speckle-tracking analysis of both ventricles and atria. Carotid ultrasonography was also performed.

All echocardiographic studies—both conventional and STE-based analyses (left ventricular, right ventricular, left atrial, and right atrial strain)—as well as carotid examinations were carried out by the same experienced cardiologist (A.S.) on the same day. Image acquisition and subsequent offline strain analysis were performed with the operator blinded to clinical data and to group assignment.

The study protocol received approval from the Comitato Etico Territoriale Lombardia 5 (reference number 597/24, with definitive approval granted on 27 February 2025).

### 2.2. Conventional Transthoracic Echocardiography

All transthoracic echocardiographic studies were carried out using a commercially available ultrasound system (Philips Sparq, Philips Medical Systems, Andover, MA, USA) equipped with a 2.5-MHz phased-array transducer. Examinations were performed with patients positioned in the left lateral decubitus position, and all measurements were obtained in accordance with the recommendations of the American Society of Echocardiography and the European Association of Cardiovascular Imaging [33,34].

Standard two-dimensional and Doppler parameters were recorded. Diameters of the aortic root and ascending aorta were measured using the leading edge-to-leading edge method. Relative wall thickness (RWT) was calculated as 2 × posterior wall thickness divided by LV end-diastolic internal diameter. Left ventricular mass index (LVMi) was derived using the Devereux formula. Left ventricular end-diastolic and end-systolic volumes were indexed to body surface area (LVEDVi and LVESVi), and left ventricular ejection fraction (LVEF) was calculated using the biplane modified Simpson method as a measure of LV systolic function [33]. Additional measurements included left atrial volume index (LAVi), right ventricular inflow tract (RVIT) diameter, tricuspid annular plane systolic excursion (TAPSE) as an indicator of right ventricular systolic performance, and inferior vena cava (IVC) diameter assessed during quiet respiration.

Doppler evaluation included assessment of the mitral E/A ratio and tissue Doppler-derived average e′ velocity; the E/average e′ ratio was calculated as an estimate of LV filling pressures [34]. Systolic pulmonary artery pressure (sPAP) was estimated using the modified Bernoulli equation as 4 × (tricuspid regurgitation velocity [TRV])^2^ plus estimated right atrial pressure, with the latter derived from IVC diameter and its respiratory collapsibility [35]. Valvular heart disease was graded according to current AHA/ACC recommendations for the management of valvular heart disease [36].

### 2.3. Speckle-Tracking Echocardiography

Following conventional transthoracic echocardiography, 2D-STE was performed using high-quality grayscale images. LV longitudinal strain analysis was obtained from apical four-, two-, and three-chamber views, while circumferential strain was derived from basal, mid-ventricular, and apical short-axis views. Analyses were conducted using automated function imaging and the Q-Analysis module (Philips QLAB) [15].

According to Philips QLAB segmentation conventions, the LV myocardium was divided into seven segments for each apical view. Peak systolic strain was defined as the maximal myocardial shortening during systole for both longitudinal and circumferential components. Global values for LV-GLS and LV global circumferential strain (LV-GCS) were displayed in a bull’s-eye plot summarizing segmental and overall strain data. Early diastolic strain rate values were extracted from longitudinal and circumferential strain curves [15].

Right ventricular global longitudinal strain (RV-GLS) was calculated from the apical four-chamber view as the mean of individual segmental strain curves. Right ventricular free-wall longitudinal strain (RV-FWLS) was computed as the average of basal, mid, and apical free-wall segments, excluding septal segments [37].

Left atrial (LA) strain analysis was performed using a biplane approach. The software automatically divided the atrial myocardium into seven segments per view. The following indices were measured: peak positive longitudinal strain (left atrial conduit strain, LAScd), peak negative longitudinal strain (left atrial contractile strain, LASct), and their sum, defined as left atrial reservoir strain (LASr). Mean values were obtained by averaging measurements from four- and two-chamber views. Strain-rate analysis provided the following curves: global positive strain rate during ventricular systole, early-diastolic strain rate, and late-diastolic strain rate [38]. An index of left atrial stiffness was calculated as LASr divided by E/average e′ [39].

Right atrial reservoir strain (RASr) was assessed by positioning fiducial markers at the tricuspid annular insertion points and along the endocardial border of the superior right atrium.

Reference values used for comparison were: LV-GLS more negative than −20% [15]; LV-GCS more negative than −23.3% [40]; RV-GLS more negative than −20% [41]; LASr greater than 39% [42]; and RASr greater than 35% [43].

### 2.4. Carotid Ultrasonography

Carotid ultrasonography was performed using the same Philips Sparq ultrasound system with a 12-MHz linear-array transducer, following a standardized imaging protocol [44]. Examinations were conducted with patients in the supine position, with slight neck extension and contralateral head rotation to optimize visualization of the carotid arteries. Image acquisition was synchronized to end-diastole, identified by the R-wave on electrocardiography. Manual measurements included the average intima-media thickness (IMT) of both left and right common carotid arteries (CCAs) and the mean end-diastolic internal diameter (CCA-EDD) of each CCA. Measurements were obtained at the distal segment of the CCA, approximately 1 cm proximal to the carotid bifurcation. Carotid relative wall thickness was calculated as 2 × average IMT divided by average CCA-EDD. The average cross-sectional area (CSA, mm^2^) of the CCA was calculated as [π × (2 × average IMT + average CCA-EDD)/2]^2^ − [π × (average CCA-EDD/2)^2^], and was used as an index of carotid arterial mass.

### 2.5. Endpoint Definition

The primary objective of the study was to evaluate whether LV-GLS, LV-GCS, RV-GLS, LASr, and RASr were reduced in patients with ECS compared with matched controls. The secondary objective was to determine predictors of subclinical left ventricular dysfunction, defined as an LV-GLS value less negative than −20%. This cutoff was chosen in accordance with contemporary recommendations from the European Association of Cardiovascular Imaging and the American Society of Echocardiography, which identify normal LV-GLS values as approximately −20%, while recognizing potential variability related to age, sex, loading conditions, and vendor-specific software platforms [15].

### 2.6. Statistical Analysis

Sample size estimation was performed a priori and indicated that inclusion of 30 ECS patients and 30 controls matched for age, sex, and cardiovascular risk factors would yield 80% statistical power to detect a 2-percentage-point difference in GLS magnitude (−20% versus −18%) at baseline, assuming a standard deviation of 2.5 in each group and applying a two-sided equal-variance t test with a significance level of α = 0.05.

The distribution of continuous variables was evaluated using the Kolmogorov–Smirnov test. Data following a normal distribution are reported as mean ± standard deviation, whereas non-normally distributed variables are presented as median (range). Comparisons between groups were performed using the independent two-tailed t test for normally distributed variables and the Mann–Whitney U test for non-parametric variables. Categorical data were analyzed using the chi-square test.

Univariate and multivariate logistic regression analyses were conducted to identify independent determinants of LV-GLS impairment (defined as LV-GLS less negative than −20% [15]) within the entire ECS cohort. Consistent with the one-in-ten rule to prevent model overfitting, the number of candidate predictors was restricted to four prespecified variables: male sex (demographic factor), time since diagnosis (disease duration), neutrophil-to-lymphocyte ratio (marker of systemic inflammation), and systolic blood pressure (global cardiovascular risk indicator). Odds ratios (ORs) with corresponding 95% confidence intervals (CIs) were calculated, and variables reaching statistical significance in univariate analysis were subsequently entered into the multivariable model.

Receiver operating characteristic (ROC) curve analysis was performed to evaluate the diagnostic performance of significant continuous predictors for the secondary endpoint of subclinical LV-GLS impairment. The area under the curve (AUC) was computed, and optimal threshold values were determined using the Youden index, defined as sensitivity + (1 − specificity).

Reproducibility of LV-GLS measurements was assessed in a randomly selected subset of 15 ECS patients who underwent repeated strain analysis by the initial echocardiographer (A.S.) and by a second independent cardiologist (M.B.), both blinded to previous results. Intra- and inter-observer agreement were quantified using the intraclass correlation coefficient (ICC) with 95% confidence intervals; ICCs ≥ 0.70 were considered indicative of acceptable reproducibility. Reproducibility testing was confined to LV-GLS because this parameter served as the predefined marker of subclinical myocardial dysfunction and the principal strain variable used in regression and ROC analyses. It represents the most extensively validated and prognostically established deformation index in systemic inflammatory disorders, including sarcoidosis.

All statistical computations were performed using SPSS version 28 (SPSS Inc., Chicago, IL, USA), and two-sided *p* values < 0.05 were considered statistically significant.

## 3. Results

### 3.1. Clinical Findings

**Figure 1:**
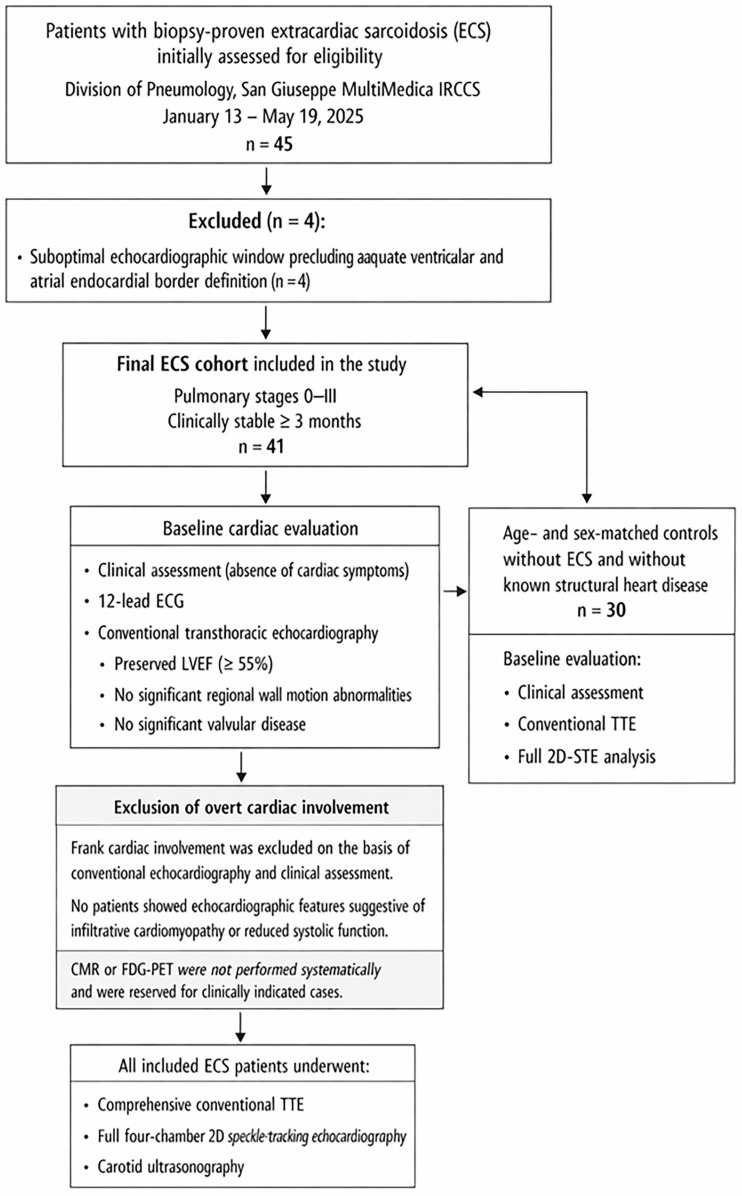
Flow-chart illustrating patient selection and cardiac assessment strategy.

The study flow diagram is presented in Figure 1.

A total of 45 consecutive patients with biopsy-proven ECS were initially assessed for eligibility at the Division of Pneumology between 13 January and 19 May 2025. Four patients were excluded due to suboptimal echocardiographic windows precluding adequate ventricular and atrial endocardial border definition. The remaining 41 patients constituted the final ECS cohort included in the study.

Overt cardiac involvement was excluded on the basis of clinical evaluation and conventional transthoracic echocardiography. In particular, no patients showed reduced left ventricular ejection fraction (LVEF ≥ 55% in all cases), significant regional wall motion abnormalities, significant valvular disease, or echocardiographic features suggestive of infiltrative cardiomyopathy. Advanced imaging techniques, such as CMR or FDG-PET, were not performed systematically and were reserved for clinically indicated cases.

We prospectively enrolled 41 patients with pulmonary sarcoidosis and compared them with 30 age- and sex-matched controls without ECS or known structural heart disease (age 57.4 ± 10.2 vs. 58.5 ± 11.1 years, *p* = 0.67; males 58.5% vs. 53.3%, *p* = 0.85).

Demographic, anthropometric, clinical, biochemical, and hemodynamic variables at baseline are reported in Table 1.

Patients with sarcoidosis showed a moderate prevalence of smoking, hypertension, and dyslipidemia and a low prevalence of type 2 diabetes, yielding a cardiovascular risk profile comparable with that of controls (all *p* > 0.05). No participant had a prior history of coronary artery disease or cerebrovascular events. Most sarcoidosis cases were stage II (56.1%), followed by stage I (26.8%) and stage III (17.1%); median disease duration was 7 years (range 1–20).

Spirometry indicated mild functional impairment: forced vital capacity (FVC) and forced expiratory volume in one second (FEV_1_) were slightly reduced yet near the lower limit of normal; mean FEV_1_/FVC was 76%, suggesting no major airflow obstruction on average. Total lung capacity (TLC) and DLCO were mildly decreased. A normal ventilatory pattern was present in 53.6% of patients, whereas 21.9% had an obstructive, 12.2% a restrictive, and 2.4% a mixed pattern. Diffusing capacity was impaired in 24.4% of cases. On the 6MWT, patients covered 480.2 ± 93.9 m with a mean oxygen desaturation of 4.0 ± 2.9%.

Laboratory testing showed no group differences in hemoglobin, systemic inflammation indices (RDW, NLR), renal function (serum creatinine, eGFR), or metabolic parameters (glucose, total cholesterol). Non-pulmonary comorbidities (cancer, COPD, OSAS, GERD, rheumatoid arthritis, hypothyroidism, mixed anxiety–depressive disorder) were likewise similar between groups. Resting heart rate and blood pressure were within the normal range and did not differ significantly.

At baseline, 34% of patients were receiving corticosteroids or inhaled therapy, and 7% were on immunosuppressants; one patient (2.4%) required oxygen therapy. Rates of cardioprotective and systemic medications were comparable, except for statins, which were more common in controls (40% vs. 15%, *p* = 0.02).

### 3.2. Conventional Echocardiographic and Carotid Ultrasound Parameters

None of the enrolled patients showed echocardiographic or clinical evidence of overt cardiac sarcoidosis at baseline evaluation. Transthoracic echocardiography showed normal LV wall thickness, normal LVMi, normal chamber sizes, and preserved biventricular systolic function (LVEF and TAPSE) in sarcoidosis patients. LV geometry was normal in 70.7%, with concentric remodeling, eccentric hypertrophy, and concentric hypertrophy in 17.1%, 7.3%, and 4.9%, respectively. First-degree diastolic dysfunction was the most frequent transmitral filling pattern, and the E/average e′ ratio commonly fell within the gray zone (8–13) [34]. Valvular disease was not clinically significant, and pulmonary hemodynamics were normal. Between-group comparisons revealed no significant differences in cardiac structure, valvular abnormalities, biventricular systolic indices, or sPAP; only the E/average e′ ratio was higher in the sarcoidosis group, suggesting subclinical elevation of LV filling pressures.

On carotid ultrasound, CCA-EDD and CCA-IMT were similar overall, with a trend toward higher IMT in sarcoidosis (0.99 vs. 0.92 mm, *p* = 0.09). Carotid relative wall thickness was greater in the sarcoidosis group (0.26 vs. 0.24, *p* = 0.04), while CCA-CSA tended to be larger without reaching significance. Although values remained within the mild range, carotid stenosis severity was greater in sarcoidosis on both the left (30% vs. 22.5%, *p* = 0.001) and right sides (28.6% vs. 23.3%, *p* = 0.02) (Table 2).

### 3.3. Speckle-Tracking Echocardiography–Derived Myocardial Strain Parameters

Table 3 summarizes regional and global biventricular and biatrial mechanics by STE.

LV mechanics showed a significant reduction in LV-GLS in sarcoidosis (−18.1% vs. −20.4%, *p* = 0.001) with impairment across the apical four-, two-, and three-chamber views. A larger proportion of patients had LV-GLS less negative than −20% (80.5% vs. 20.0%, *p* < 0.001). Global longitudinal strain rate was reduced in all views. LV-GCS was lower in sarcoidosis (median −20.2% vs. −24.1%, *p* < 0.001), mainly due to reduced basal and mid-ventricular circumferential strain, with apical values preserved. More patients had a GCS less negative than −23.3%, but the difference was not significant (19.5% vs. 10.0%, *p* = 0.34). Global circumferential strain rate was also lower (−1.34 vs. −1.60 s^−1^, *p* < 0.001).

Left atrial mechanics were significantly impaired in patients with sarcoidosis compared with controls: LAScd and LASr were reduced (22.9% vs. 28.4%, *p* < 0.001; 30.2% vs. 38.1%, *p* < 0.001, respectively), and a higher proportion of patients had abnormal LASr (<39%) (70.7% vs. 20.0%, *p* < 0.001). Strain-rate analysis further showed lower systolic and early-diastolic indices, together with a reduced LASr/E/e′ ratio (3.56 vs. 4.58%, *p* = 0.003), consistent with impaired LA function and elevated LV filling pressures in sarcoidosis.

Right ventricular mechanics were also markedly abnormal in sarcoidosis patients: free-wall longitudinal strain (RV-FWLS, −17.5% vs. −24.6%, *p* < 0.001) and global longitudinal strain (RV-GLS, −16.8% vs. −23.5%, *p* < 0.001) were significantly reduced, with abnormal RV-GLS (less negative than −20%) observed in 85.4% of patients versus only 6.7% of controls (*p* < 0.001). RV strain-rate indices were likewise decreased. Right atrial (RA) reservoir and conduit strain were impaired (RASr 28.5% vs. 35.2%, *p* < 0.001; RAScd 21.6% vs. 25.5%, *p* = 0.02), and abnormal RASr (<35%) was substantially more frequent in patients (68.3% vs. 16.7%, *p* < 0.001). RA strain-rate analysis corroborated the presence of functional impairment.

The time required for strain analysis was slightly but significantly longer in sarcoidosis (9.1 ± 1.5 vs. 8.0 ± 1.8 min, *p* = 0.009).

**Figure 2:**
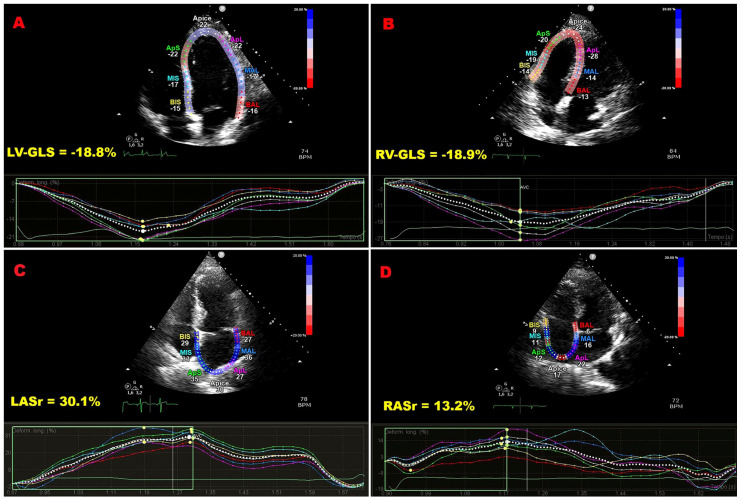
Illustrative example of LV-GLS (**A**), RV-GLS (**B**), LASr (**C**), and RASr (**D**) assessment by speckle tracking echocardiography in an ECS patient participating in the present study. ECS, extracardiac sarcoidosis; GLS, global longitudinal strain; LASr, left atrial reservoir strain; LV, left ventricular; RASr, right atrial reservoir strain; RV, right ventricular.

**Figure 3:**
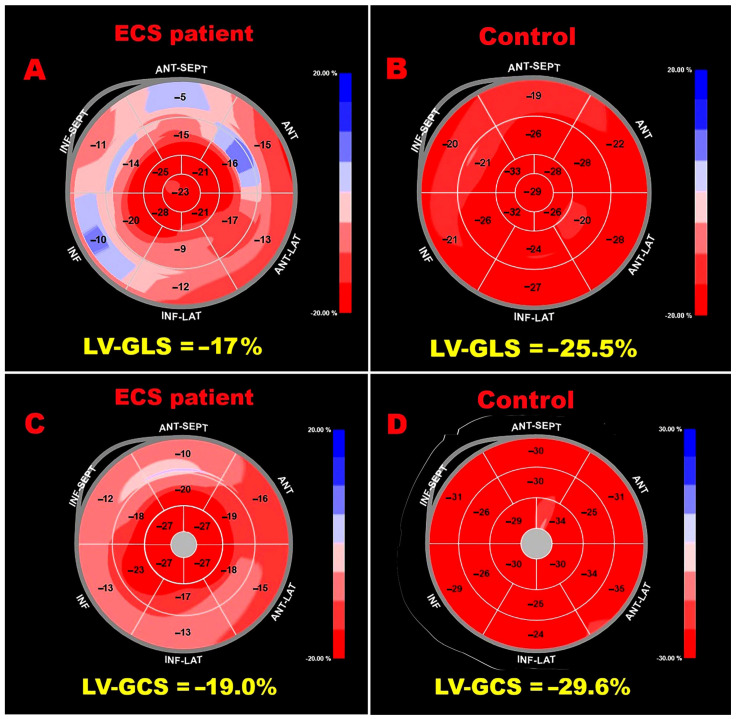
Bull’s eye plots derived from 2D-STE illustrating LV-GLS and LV-GCS in an ECS patient ((**A**) and (**C**), respectively) and in a control ((**B**) and (**D**), respectively). 2D, two-dimensional; ECS, extracardiac sarcoidosis; GCS, global circumferential strain; GLS, global longitudinal strain; LV, left ventricular; STE, speckle tracking echocardiography.

Figure 2 shows representative LV-GLS, RV-GLS, LASr, and RASr assessments in an ECS patient, and Figure 3 displays bull’s-eye plots of LV-GLS and LV-GCS for an ECS patient and a control without ECS.

### 3.4. Variability Analysis

Reproducibility was high. Intra-observer agreement was excellent for LV-GLS and LV-GCS (ICC 0.93, 95% CI 0.81–0.98; and 0.92, 95% CI 0.80–0.97). Inter-observer reproducibility was good (LV-GLS ICC 0.82, 95% CI 0.54–0.93; LV-GCS ICC 0.83, 95% CI 0.56–0.94) (Table 4).

### 3.5. Univariate and Multivariate Logistic Regression

On univariate analysis, male sex was associated with higher odds of LV-GLS impairment (OR 6.00, 95% CI 1.04–34.7, *p* = 0.046), but this was not independent after adjustment (OR 3.04, 95% CI 0.26–35.5, *p* = 0.37). NLR showed a significant univariate association (OR 1.10, 95% CI 1.01–1.20, *p* = 0.025) that attenuated in the multivariable model (OR 1.12, 95% CI 0.98–1.28, *p* = 0.10). In contrast, years since diagnosis remained an independent predictor: each additional year more than doubled the odds of LV-GLS impairment in both univariate (OR 2.27, 95% CI 1.26–4.09, *p* = 0.006) and multivariate analyses (OR 2.26, 95% CI 1.10–4.65, *p* = 0.03). Systolic blood pressure was not associated with the outcome (OR 1.01, 95% CI 0.96–1.06, *p* = 0.62) (Table 5).

**Figure 4:**
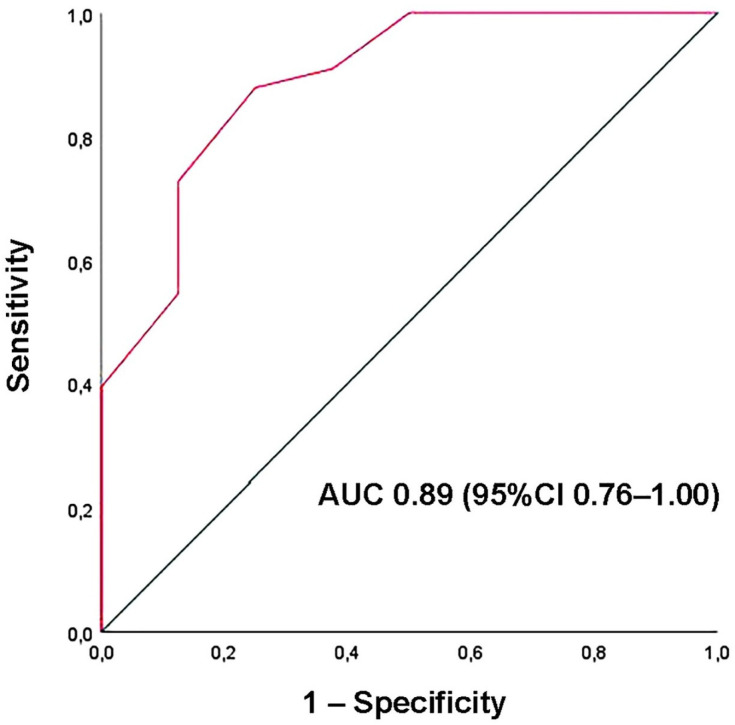
ROC curve analysis performed to determine the optimal cutoff for disease duration in predicting impaired LV-GLS within the ECS cohort. ECS, extracardiac sarcoidosis; GLS, global longitudinal strain; LV, left ventricular; ROC, Receiver operating characteristic.

Receiver-operating characteristic analysis identified a disease duration of 4.5 years as the optimal cutoff to predict LV-GLS impairment, with 88% sensitivity and 75% specificity (AUC = 0.89; 95% CI 0.76–1.00) (Figure 4).

## 4. Discussion

### 4.1. Main Findings of the Present Study

In this prospective cohort of ECS patients with only mild pulmonary involvement, conventional TTE excluded overt infiltrative cardiomyopathy while showing mildly increased LV filling pressures, consistent with early subclinical LV diastolic dysfunction. Despite preserved conventional systolic parameters (LVEF and TAPSE), STE identified a diffuse pattern of myocardial impairment, with significant reductions in LV global deformation in both longitudinal and circumferential directions, as well as decreased RV free-wall and global strain. These findings demonstrate early biventricular mechanical dysfunction that would have remained undetected using standard echocardiographic indices alone.

LV-GCS reduction predominantly affected the basal and mid-ventricular segments, with relative preservation of apical function, suggesting an apical-sparing pattern. This distribution may reflect compensatory recruitment of apical mechanics in response to early regional myocardial involvement. In parallel, biatrial function was significantly impaired, with lower LA and RA reservoir and conduit strain values. These abnormalities likely represent the combined influence of elevated LV filling pressures, myocardial stiffening, and potential direct atrial myocardial involvement.

Multivariable analysis identified years since diagnosis as the only independent predictor of LV-GLS impairment (less negative than −20%), indicating that myocardial deformation abnormalities are more closely associated with disease duration than with resting hemodynamics or systemic inflammatory burden. This temporal association supports the concept of progressive and cumulative myocardial involvement in ECS, even among clinically stable patients without overt cardiac manifestations.

In addition, despite a similar traditional cardiovascular risk profile, ECS patients exhibited greater carotid relative wall thickness and more severe carotid stenosis compared with controls, suggesting early and possibly accelerated vascular remodeling. Taken together with the myocardial strain findings, these vascular alterations point toward a broader inflammatory and cardiometabolic phenotype in ECS extending beyond the myocardium.

### 4.2. Comparison with Previous Studies and Interpretation of Results

Over the past decade, several echocardiographic studies have examined patients with ECS lacking overt heart disease using conventional 2D-TTE complemented by 2D-STE, with the goal of detecting early, subclinical myocardial dysfunction. These cohorts typically featured a high prevalence of pulmonary involvement, preserved cardiac structure and LVEF on TTE, and predominantly female participants with low-to-moderate rates of standard cardiovascular risk factors. Across studies, STE consistently revealed subclinical LV mechanical impairment despite normal LVEF. A recent meta-analysis from our group that pooled 13 studies (785 sarcoidosis patients and 567 controls) showed that sarcoidosis exerts a larger effect on LV-GLS than on LVEF [45]. This LV-GLS reduction was robust to multiple potential confounders, including age, sex, smoking, hypertension, type 2 diabetes, dyslipidemia, disease duration, and ultrasound platform. In the subset with both echocardiography and CMR, impaired LV-GLS correlated with the extent of myocardial inflammation/fibrosis on LGE [16,17,20]. Approximately half of the included studies further linked LV-GLS to adverse outcomes—overall mortality, incident heart failure, arrhythmias, device implantation, or subsequent development of cardiac sarcoidosis—over mid-term follow-up.

Our findings are consistent with this body of evidence. We observed normal chamber dimensions, preserved biventricular systolic function, mild diastolic dysfunction with relatively higher LV filling pressures, and normal pulmonary hemodynamics on conventional TTE, yet widespread subclinical abnormalities on STE. Extending prior work that has largely focused on LV longitudinal mechanics, we performed a systematic four-chamber deformation assessment and documented mild but significant attenuation of biventricular and biatrial strain indices. This integrated approach suggests that myocardial involvement in ECS may represent a diffuse, multi-chamber mechanical phenotype rather than isolated LV impairment.

Importantly, apical circumferential strain remained relatively preserved compared with basal and mid-ventricular segments, suggesting a relative apical sparing pattern even at subclinical stages of cardiac involvement. While classically associated with cardiac amyloidosis [46], apical sparing has also been described in hypertrophic cardiomyopathy, hypertensive LV hypertrophy, end-stage renal disease, coronary disease (particularly right coronary or left circumflex territories), reverse Takotsubo syndrome, chemotherapy-related cardiomyopathy, and mitral valve prolapse [47,48,49]. Preservation of apical circumferential mechanics may help maintain a normal LVEF despite impaired longitudinal shortening, underscoring why LV-GLS appears more sensitive than LVEF for early dysfunction detection [50].

In our cohort, LV-GLS impairment was more closely associated with disease duration than with resting hemodynamic parameters or systemic inflammatory indices, suggesting a potential cumulative effect of chronic inflammatory exposure on myocardial mechanics. Although causal inferences cannot be drawn from this cross-sectional design, these findings support the rationale for early detection and longitudinal surveillance of myocardial function in patients with ECS.

To our knowledge, this is the first study to combine detailed regional LV strain and strain-rate analysis in both longitudinal and circumferential directions with comprehensive right ventricular (global and free-wall) and biatrial systolic and diastolic strain assessment in ECS patients without fibrotic pulmonary involvement. Beyond the incremental diagnostic yield relative to conventional echocardiographic parameters, our reproducibility analysis supports the clinical reliability of STE-derived LV metrics.

Although atherosclerosis is not traditionally considered a hallmark of sarcoidosis, our vascular findings suggest a potential association between systemic inflammation and early vascular remodeling. Consistent with Yilmaz et al. [51], who demonstrated increased carotid IMT and impaired flow-mediated dilation in sarcoidosis—indicative of endothelial dysfunction and subclinical atherosclerosis—we observed carotid remodeling despite comparable traditional cardiovascular risk profiles. Notwithstanding the anti-inflammatory and anti-atherosclerotic properties of statins [52,53], these agents appeared relatively underused in our sarcoidosis cohort, a finding that may warrant further investigation in larger prospective studies.

### 4.3. Pathophysiological Mechanisms Underpinning Subclinical Myocardial Dysfunction in Sarcoidosis Patients

Cardiac sarcoidosis primarily affects the myocardium, whereas pericardial and endocardial involvement generally occurs as an extension of myocardial lesions [54]. The characteristic histopathological features include patchy non-caseating granulomas, lymphocytic infiltration, interstitial edema, and enhanced fibrotic remodeling, typically distributed in a focal and heterogeneous pattern within the myocardium [55]. The most commonly involved regions, in decreasing order of frequency, are the LV free wall, the interventricular septum, the papillary muscles, the RV, and the atria [56].

Reduction in LV-GLS is believed to reflect the tendency of granulomatous inflammation to involve predominantly the mid-myocardial layer, which plays a pivotal role in longitudinal fiber shortening [57]. The inflammatory process promotes progressive fibrosis and scar formation, resulting in attenuation of myocardial deformation. Given the patchy and initially localized nature of myocardial involvement, early disease is more likely to manifest as impaired LV-GLS rather than reduced LVEF, thereby explaining why conventional echocardiographic parameters may remain within normal limits in the initial stages. With progression toward more transmural involvement, impairment of circumferential mechanics, reflected by reductions in LV-GCS, is anticipated.

RV dysfunction may arise through several mechanisms. Decreased RV-GLS may be attributable to direct granulomatous infiltration of the RV free wall, to increased sPAP secondary to pulmonary involvement, or to chronic exposure to elevated LV filling pressures related to LV diastolic dysfunction [18].

The observed decline in biatrial reservoir function in ECS patients likely reflects either primary atrial myocardial involvement or sustained elevation of LV filling pressures, or a combination of both mechanisms [58,59].

In the present cohort, the relatively low prevalence of traditional cardiovascular risk factors, the absence of prior CAD and significant valvular disease, together with the findings of logistic regression analysis, support the interpretation that the detected subclinical myocardial abnormalities are predominantly related to sarcoidosis and its hemodynamic consequences rather than to confounding comorbid conditions.

### 4.4. Implications for Clinical Practice

To the best of our knowledge, this investigation represents the first prospective study to provide a systematic four-chamber assessment of myocardial deformation in ECS patients without fibrotic pulmonary disease. In contrast to most previous STE studies in ECS, which have predominantly centered on LV longitudinal strain and only sporadically examined RV function, our analysis encompassed deformation parameters of both ventricles and both atria. This comprehensive approach enables a broader depiction of the myocardial mechanical profile in ECS and underscores diffuse, multi-chamber subclinical dysfunction rather than isolated LV involvement. These observations support the potential utility of STE as a bridge between conventional echocardiography and more advanced cardiac imaging techniques. Incorporating LV-GLS into routine echocardiographic evaluation may enhance early detection of subtle myocardial abnormalities and help identify patients who may warrant further assessment with CMR or FDG-PET, even when conventional systolic indices remain preserved.

From a clinical standpoint, 2D-STE provides additional diagnostic insight beyond standard 2D-TTE and may be particularly helpful when advanced imaging modalities are not readily accessible or are contraindicated. In ECS patients reporting palpitations or presenting with non-specific ECG changes, a preserved LV-GLS (more negative than −20%) may reduce the likelihood of early cardiac involvement. In contrast, a less negative LV-GLS should prompt intensified cardiologic follow-up and consideration of further diagnostic evaluation. When deformation abnormalities are identified, multimodality imaging becomes especially relevant for risk stratification. CMR with LGE provides a detailed evaluation of myocardial fibrosis and scar distribution, while FDG-PET enables detection of active inflammation. Combining STE-derived functional data with structural and metabolic information from CMR and/or FDG-PET may improve overall diagnostic precision and help refine risk assessment in ECS, as supported by prior literature [60].

Beyond its diagnostic value, LV-GLS may also carry prognostic implications. Reduced LV-GLS has been associated with an increased incidence of adverse cardiac events in sarcoidosis over intermediate-term follow-up [24,25], suggesting that patients with impaired deformation may benefit from closer surveillance. It is conceivable—although not yet demonstrated—that early identification of LV-GLS abnormalities in ECS could allow timely therapeutic intervention aimed at preventing progression to overt CS and mitigating the risk of arrhythmias or conduction disturbances. In this context, earlier initiation of immunosuppressive therapy might theoretically be more effective before the development of manifest systolic dysfunction [61,62,63]. Nonetheless, these considerations remain speculative and require validation in dedicated prospective outcome studies.

Finally, the evidence of early subclinical atherosclerotic changes in ECS reinforces the importance of proactive cardiovascular risk assessment and aggressive management of modifiable risk factors. In light of our findings, earlier introduction of statin therapy after diagnosis may merit consideration, particularly given their anti-inflammatory and vascular-protective properties [52,53].

### 4.5. Study Limitations

Several limitations of the present study should be acknowledged. First, it was conducted at a single center and included a relatively limited number of participants, although the sample size was supported by an a priori power calculation. The ECS population was heterogeneous in terms of disease duration, pulmonary functional patterns, and ongoing respiratory treatment. Moreover, the absence of systematic advanced imaging—such as CMR or FDG-PET—prevented direct correlation of echocardiographic strain abnormalities with objective markers of myocardial inflammation or fibrosis, which represents a relevant methodological constraint. Biomarkers potentially informative for myocardial involvement, including C-reactive protein and N-terminal pro-B-type natriuretic peptide, were not available. In addition, strain imaging is subject to technical limitations, including inter-vendor variability, operator dependence, image quality constraints, frame-rate optimization (with low frame rates potentially leading to speckle dropout), sensitivity to loading conditions, and external mechanical influences such as chest wall configuration, all of which may affect measurement accuracy [64,65,66,67].

Furthermore, the cross-sectional design and lack of longitudinal follow-up data limit the ability to draw conclusions regarding the prognostic significance of the detected strain abnormalities. As a result, any consideration of the potential prognostic or therapeutic implications of impaired LV-GLS should be regarded as extrapolated from existing literature and interpreted as hypothesis-generating rather than definitive. Future prospective studies with longitudinal follow-up are required to determine whether subclinical myocardial dysfunction identified by 2D-STE is associated with adverse clinical outcomes in ECS patients.

## 5. Conclusions

In this single-center prospective study, patients with ECS without fibrotic pulmonary involvement exhibited subclinical impairment of biventricular and biatrial strain indices despite preserved conventional systolic function. These alterations were associated with longer disease duration, suggesting a potential link between chronic inflammatory burden and myocardial mechanical dysfunction.

While the cross-sectional design precludes causal or prognostic inferences, our findings support the potential role of comprehensive four-chamber 2D-STE as a sensitive tool for detecting early myocardial involvement in ECS. Larger, multicenter studies incorporating systematic advanced cardiac imaging and longitudinal outcome data are warranted to better define the clinical and prognostic significance of these strain abnormalities and to determine whether they may inform patient management strategies.

## Figures and Tables

**Table 1:** Baseline demographic, clinical, biochemical, and hemodynamic characteristics of ECS patients and controls.

Clinical Variables	Sarcoidosis Patients (n = 41)	Controls (n = 30)	*p* Value
Demographics and anthropometrics			
Age (yrs)	57.4 ± 10.2	58.5 ± 11.1	0.67
Male sex (%)	24 (58.5)	16 (53.3)	0.85
BSA (m^2^)	1.96 ± 0.23	1.92 ± 0.13	0.36
BMI (Kg/m^2^)	27.9 ± 4.9	26.3 ± 3.5	0.11
**Cardiovascular risk factors**			
Smoking (%)	13 (31.7)	10 (33.3)	0.89
Hypertension (%)	18 (43.9)	15 (50.0)	0.61
Type 2 diabetes (%)	4 (9.7)	7 (23.3)	0.18
Dyslipidemia (%)	11 (26.8)	8 (26.7)	0.99
**Clinical staging**			
I (%)	11 (26.8)	/	/
II (%)	23 (56.1)	/	/
III (%)	7 (17.1)	/	/
Yrs from diagnosis	7.2 (1–20)	/	/
**Spirometry parameters**			
FVC (l)	3.7 ± 1.1	/	/
FVC (%)	91.3 ± 14.4	/	/
FEV (l)	2.79 (1.21–6.04)	/	/
FEV_1_ (%)	88.4 ± 17.6	/	/
FEV_1_/FVC ratio	76.1 ± 8.9	/	/
TLC (l)	5.5 ± 1.4	/	/
TLC (%)	88 (50–116)	/	/
DLCO (ml/min/mmHg)	6.7 ± 1.5	/	/
DLCO (%)	81.8 ± 14.1	/	/
Normal pattern (%)	22 (53.6)	/	/
Obstructive pattern (%)	9 (21.9)	/	/
Restrictive pattern (%)	5 (12.2)	/	/
Mixed pattern (%)	1 (2.4)	/	/
Impaired diffusion capacity (%)	10 (24.4)	/	/
6MWT (m)	480.2 ± 93.9	/	/
ΔSaO_2_ (%)	4.0 ± 2.9	/	/
**Blood tests**			
Serum hemoglobin (g/dL)	13.6 ± 1.3	13.7 ± 1.2	0.74
RDW (%)	13.3 ± 0.9	13.1 ± 0.8	0.33
NLR	0.63 ± 0.14	0.59 ± 0.12	0.20
Serum creatinine (mg/dL)	0.85 ± 0.17	0.90 ± 0.14	0.18
eGFR (ml/min/1.73 m^2^)	87.9 ± 18.4	93.8 ± 15.5	0.15
Serum glucose (mg/dL)	95.5 ± 11.4	100.1 ± 18.2	0.23
Serum total cholesterol (mg/dL)	196.6 ± 52.7	204.1 ± 43.4	0.51
**Comorbidities**			
Cancers (%)	1 (2.4)	2 (6.7)	0.57
COPD (%)	3 (7.3)	1 (3.3)	0.63
OSAS (%)	3 (7.3)	1 (3.3)	0.63
GERD (%)	6 (14.6)	6 (20.0)	0.74
Rheumatoid arthritis (%)	4 (9.7)	1 (3.3)	0.39
Hypothyroidism (%)	4 (9.7)	4 (13.3)	0.71
Mixed anxiety–depressive disorder (%)	1 (2.4)	3 (10.0)	0.32
**Hemodynamics**			
Heart rate (bpm)	72.1 ± 12.2	69.0 ± 8.4	0.23
SBP (mmHg)	129.8 (104–190)	132.5 (105–165)	0.46
DBP (mmHg)	77.6 (63–100)	79.3 (65–100)	0.45
**Respiratory treatment**			
Oxygen therapy (%)	1 (2.4)	/	/
Oral corticosteroids (%)	14 (34.1)	/	/
Inhalation therapy (%)	14 (34.1)	/	/
Immunosuppressants (%)	3 (7.3)	/	/
**Nonrespiratory treatment**			
Antiplatelets (%)	4 (9.7)	4 (13.3)	0.72
Anticoagulants (%)	1 (2.4)	2 (6.7)	0.57
ACEi-ARBs (%)	13 (31.7)	11 (36.7)	0.66
Calcium channel blockers (%)	6 (14.6)	6 (20.0)	0.57
Beta blockers (%)	11 (26.8)	12 (40.0)	0.25
Diuretics (%)	6 (14.6)	7 (23.3)	0.37
Statins (%)	6 (14.6)	12 (40.0)	**0.02**
Hydroxychloroquine (%)	4 (9.7)	1 (3.3)	0.39
Oral hypoglycaemic agents (%)	4 (9.7)	7 (23.3)	0.11
Proton pump inhibitors (%)	6 (14.6)	6 (20.0)	0.74

Normally distributed data are presented as mean ± SD, while non-normal data are presented as median (range). Categorical variables are expressed as percentages. Significant *p* values are in bold. ΔSaO_2_, change in oxygen saturation from stress to rest; 6MWT, Six-Minute Walk Test; ACEi/ARBs, Angiotensin-Converting Enzyme Inhibitors/Angiotensin II Receptor Blockers; BMI, body mass index; BSA, body surface area; COPD, chronic obstructive pulmonary disease; DBP, diastolic blood pressure; DLCO, diffusing capacity of the lung for carbon monoxide; ECS, extracardiac sarcoidosis; eGFR, estimated glomerular filtration rate; FEV_1_, forced expiratory volume in one second; FVC, forced vital capacity; GERD, gastroesophageal reflux disease; NLR, neutrophil-to-lymphocyte ratio; OSAS, obstructive sleep apnea syndrome; RDW, red cell distribution width; SBP, systolic blood pressure; TLC, total lung capacity.

**Table 2:** Conventional TTE and carotid ultrasound parameters obtained in ECS patients and controls at basal evaluation.

Conventional TTE Variables	Sarcoidosis (n = 41)	Controls (n = 30)	*p* Value
LV size and systolic function			
IVS thickness (mm)	11.5 ± 2.3	11.3 ± 2.6	0.74
PW thickness (mm)	8.6 ± 1.3	8.7 ± 1.5	0.77
LVEDD (mm)	47.2 ± 4.1	48.2 ± 3.6	0.28
RWT	0.37 ± 0.06	0.36 ± 0.07	0.53
LVMi (g/m^2^)	87.1 (40.2–165.8)	89.3 (48–123)	0.70
LVEDVi (ml/m^2^)	39.2 (24.2–71.9)	42.5 (29.3–58.8)	0.13
LVESVi (ml/m^2^)	13.6 (7.0–30.8)	15.2 (12.6–17.7)	0.13
LVEF (%)	65.8 (45–75)	64.2 (57–70)	0.21
**LV geometric pattern**			
Normal (%)	29 (70.7)	20 (66.7)	0.80
Concentric remodeling (%)	7 (17.1)	2 (6.7)	0.29
Eccentric hypertrophy (%)	3 (7.3)	5 (16.6)	0.27
Concentric hypertrophy (%)	2 (4.9)	3 (10.0)	0.64
**LV diastolic function**			
E/A ratio	0.87 (0.51–2.0)	0.80 ± 0.20	0.26
E/average e’ ratio	10.1 (6.0–21.5)	8.5 (4.5–12.4)	**0.03**
**LA size**			
LA A-P diameter (mm)	40.8 (27.5–62.0)	40.4 (35–46)	0.73
LA longitudinal diamerer (mm)	51.7 (40.0–89.0)	50.3 (42–55)	0.37
LAVi (ml/m^2^)	28.5 (14.8–90.6)	32.1 (22.0–43.3)	0.16
**Valvular regurgitation**			
More than mild MR (%)	2 (4.9)	3 (13.3)	0.64
More than mild AR (%)	1 (2.4)	3 (13.3)	0.30
More than mild TR (%)	5 (12.2)	5 (16.6)	0.73
**RV size and systolic function**			
RVIT (mm)	30.6 (24.5–52.0)	31.1 (25–37)	0.59
TAPSE (mm)	22.4 ± 3.7	22.5 ± 3.0	0.90
**Pulmonary hemodynamics**			
TRV (m/s)	2.45 (2.0–2.95)	2.47 (2.18–2.74)	0.71
sPAP (mmHg)	29.3 (21–39)	30.1 (24–40)	0.49
**Aortic dimensions**			
Aortic root (mm)	34.8 (27–49)	34.2 (29–44)	0.56
Ascending aorta (mm)	33.4 (26.5–41.0)	33.1 (28–42.5)	0.76
Aortic arch (mm)	28.3 (22.5–35.0)	27.5 (23.5–34)	0.21
**Carotid ultrasound variables**			
CCA-EDD (mm)	7.6 (6.2–9.5)	7.5 (7.0–8.3)	0.49
CCA-IMT (mm)	0.99 (0.55–1.50)	0.92 (0.60–1.10)	0.09
Carotid RWT	0.26 (0.18–0.32)	0.24 (0.16–0.30)	**0.04**
Carotid CSA (mm^2^)	26.9 (12.8–47.1)	24.4 (15.3–29.2)	0.08
Left carotid artery stenosis (%)	30 (15–45)	22.5 (10–40)	**0.001**
Right carotid artery stenosis (%)	28.6 (20–45)	23.3 (10–45)	**0.02**

Normally distributed data are presented as mean ± SD, while non-normal data are presented as median (range). Categorical variables are expressed as percentages. Significant *p* values are in bold. A-P, antero-posterior; AR, aortic regurgitation; CCA, common carotid artery; CSA, cross-sectional area; ECS, extracardiac sarcoidosis; EDD, end-diastolic diameter; IMT, intima-media thickness; IVS, interventricular septum; LA, left atrial; LAVi, left atrial volume index; LVEDD, left ventricular end-diastolic diameter; LVEDVi, left ventricular end-diastolic volume index; LVEF, left ventricular ejection fraction; LVESVi, left ventricular end-systolic volume index; LVMi, left ventricular mass index; MR, mitral regurgitation; PW, posterior wall; RVIT, right ventricular inflow tract; RWT, relative wall thickness; sPAP, systolic pulmonary artery pressure; TAPSE, tricuspid annular plane systolic excursion; TR, tricuspid regurgitation; TRV, tricuspid regurgitation velocity; TTE, transthoracic echocardiography.

**Table 3:** STE-derived myocardial strain parameters measured in ECS patients and controls at basal evaluation.

STE Variables	Sarcoidosis (n = 41)	Controls (n = 30)	*p* Value
LV regional and global longitudinal strain			
LS 4C view (%)	−18.5 ± 2.5	−20.7 ± 2.7	**<0.001**
LS 2C view (%)	−18.4 ± 3.2	−20.5 ± 2.9	**0.005**
LS 3C view (%)	−17.4 ± 3.6	−20.1 ± 3.1	**0.001**
GLS (%)	−18.1 ± 2.6	−20.4 ± 2.9	**0.001**
LV-GLS less negative than −20% (%)	33 (80.5)	6 (20.0)	**<0.001**
**LV regional and global longitudinal strain rate**			
LSR 4C view (s^−1^)	−1.01 (–0.7,–1.3)	−1.15 (–0.97,–1.33)	**0.001**
LSR 2C view (s^−1^)	−0.98 ± 0.14	−1.13 ± 0.16	**<0.001**
LSR 3C view (s^−1^)	−1.00 (–0.6,–1.3)	−1.11 (–0.94,–1.28)	**0.01**
GLSR (s^−1^)	−1.00 ± 0.11	−1.13 ± 0.17	**<0.001**
**LV regional and global circumferential strain**			
CS at base level (%)	−16.5 ± 4.5	−21.3 ± 2.2	**<0.001**
CS at papillary muscles level (%)	−19.4 ± 4.5	−24.5 ± 2.9	**<0.001**
CS at apex level (%)	−27.2 ± 6.2	−26.5 ± 6.4	0.65
GCS (%)	−20.2 (–13.6,−29.6)	−24.1 (–20.3,–32.9)	**<0.001**
LV-GCS less negative than −23.3% (%)	8 (19.5)	3 (10.0)	0.34
**LV regional and global circumferential strain rate**			
CSR 4C view (s^−1^)	−1.14 ± 0.20	−1.37 ± 0.22	**<0.001**
CSR 2C view (s^−1^)	−1.29 ± 0.26	−1.65 ± 0.29	**<0.001**
CSR 3C view (s^−1^)	−1.80 ± 0.36	−1.78 ± 0.35	0.81
GCSR (s^−1^)	−1.34 ± 0.23	−1.60 ± 0.29	**<0.001**
**LA phasic strain**			
LAScd (%)	22.9 (5.7–36.8)	28.4 (21–37.5)	**<0.001**
LASct (%)	–7.4 (–1,–19)	–9.6 (–1,–16.4)	**0.07**
LASr (%)	30.2 (17–55.8)	38.1 (27.8–46)	**<0.001**
LASr less positive than 39% (%)	29 (70.7)	6 (20.0)	**<0.001**
**LA phasic strain rate**			
SRs (s^−1^)	1.61 (0.6–2.7)	1.98 (1.2–3.8)	**0.008**
SRe (s^−1^)	−1.65 (–0.9,–3.7)	−2.47 (–1.7,–4.2)	**<0.001**
SRl (s^−1^)	−2.44 ± 0.66	2.74 ± 0.56	**0.04**
LASr/E/average e’ ratio (%)	3.56 ± 1.34	4.58 ± 1.37	**0.003**
**RV regional and global strain**			
FWLS (%)	−17.5 (–12,–27.5)	−24.6 (–19.0,–29.6)	**<0.001**
GLS (%)	−16.8 (–11.5,–26.5)	−23.5 (–18,–28.5)	**<0.001**
RV-GLS less negative than −20% (%)	35 (85.4)	2 (6.7)	**<0.001**
**RV global strain rate**			
GLSR (s^−1^)	−1.06 ± 0.18	1.22 ± 0.19	**<0.001**
**RA phasic strain**			
RAScd (%)	21.6 ± 7.5	25.5 ± 5.8	**0.02**
RASct (%)	−6.9 (–1,–20)	−9.7 (–4,–25)	**0.02**
RASr (%)	28.5 (17–49.4)	35.2 ± 7.7	**<0.001**
RASr less positive than 35% (%)	28 (68.3)	5 (16.7)	**<0.001**
**RA phasic strain rate**			
SRs (s^−1^)	1.79 (1–3.3)	2.01 (1.21–3.51)	0.12
SRe (s^−1^)	−1.46 ± 0.41	−1.68 ± 0.44	**0.04**
SRl (s^−1^)	−1.85 ± 0.56	−2.15 ± 0.61	**0.04**
**Timing** (minutes)	9.1 ± 1.5	8.0 ± 1.8	**0.009**

Normally distributed data are presented as mean ± SD, while non-normal data are presented as median (range). Categorical variables are expressed as percentages. Significant *p* values are in bold. CS, circumferential strain; CSR 2C view, circumferential strain rate in two-chamber view; CSR 3C view, circumferential strain rate in three-chamber view; CSR 4C view, circumferential strain rate in four-chamber view; ECS, extracardiac sarcoidosis; FWLS, free-wall longitudinal strain; GCS, global circumferential strain; GCSR, global circumferential strain rate; GLS, global longitudinal strain; GLSR, global longitudinal strain rate; LAScd, left atrial strain during conduit phase; LASct, left atrial strain during contraction phase; LASr, left atrial strain during reservoir phase; LS 4C, longitudinal strain in four-chamber view; LS 2C view, longitudinal strain in two-chamber view; LS 3C view, longitudinal strain in three-chamber view; LSR 2c view, longitudinal strain rate in two-chamber view; LSR 3C view, longitudinal strain rate in three-chamber view; LSR 4C view, longitudinal strain rate in four-chamber view; RA, right atrial; RAScd, right atrial strain during conduit phase; RASct, right atrial strain during contraction phase; RASr, right atrial strain during reservoir phase; RV, right ventricular; SRe, strain rate during early diastole; SRl, strain rate during late diastole; SRs, strain rate during systole; STE, speckle tracking echocardiography.

**Table 4:** The intra- and inter-observer reproducibility of STE-based measurements of LV-GLS and LV-GCS performed in a subgroup of 15 randomly selected ECS patients.

	LV-GLS			LV-GCS		
Patient List	Initial Measurement	Remeasurements		Initial Measurement	Remeasurements	
		Rater 1	Rater 2		Rater 1	Rater 2
(1) L.B.	15.3	16	16.5	20.4	20.8	22
(2) A.D.	16.8	17	17.1	19	19.5	21
(3) A.C.	19	18	17	22.7	20.3	19.5
(4) F.N.	15.1	13.5	12	13.6	15	15.6
(5) M.L.	19.6	20	20.2	13.8	13.1	12.6
(6) M.B.	16.6	16.9	17	14.7	13.5	13
(7) C.M.	18.4	18.5	18.8	15.4	15	14.5
(8) M.F.	18	17	16.5	16.5	16	15
(9) F.B.	18.9	18	16	17	16.1	15.8
(10) L.S.	16.4	15.5	15	17.1	17	17.1
(11) S.R.	19.8	19	19	17.3	16.7	16.5
(12) G.S.	24.4	23.5	23	17.5	17	16.8
(13) M.P.	16.2	17	17.1	17.5	18	18.1
(14) P.B.	19.2	20	20.2	18	18.3	18.9
(15) D.S.	17.9	18.8	18.9	18.2	17.5	17
ICC (95%CI)		0.93 (0.81–0.98)	0.82 (0.54–0.93)		0.92 (0.80–0.97)	0.83 (0.56–0.94)

ECS, extracardiac sarcoidosis; GCS, global circumferential strain; GLS, global longitudinal strain; LV, left ventricular; STE, speckle tracking echocardiography.

**Table 5:** Univariate and multivariate logistic regression analysis identifying the independent predictors of subclinical myocardial dysfunction (defined as LV-GLS less negative than –20%) in the whole cohort of ECS patients.

	Univariate Logistic Regression Analysis			Multivariate Logistic Regression Analysis		
Variables	OR	95% CI	*p* Value	OR	95% CI	*p* Value
Male sex	6.00	1.04–34.7	0.046	3.04	0.26–35.5	0.37
Years from diagnosis	2.27	1.26–4.09	0.006	2.26	1.10–4.65	0.03
NLR	1.10	1.01–1.20	0.025	1.12	0.98–1.28	0.10
SBP (mmHg)	1.01	0.96–1.06	0.62			

ECS, extracardiac sarcoidosis; GLS, global longitudinal strain; LV, left ventricular; NLR, neutrophil-to-lymphocyte ratio; SBP, systolic blood pressure.

## Data Availability

Data extracted from included studies will be publicly available on Zenodo (https://zenodo.org).
